# Cell type specific contributions of mTOR inhibition that drive neuroprotective mechanisms in Alzheimer’s disease

**DOI:** 10.1002/alz.092635

**Published:** 2025-01-03

**Authors:** Kathryn A Haynes, Diogo Francisco Silva Santos, Sean‐Paul Gerard Williams, Jason T Hart, Alicia M Lehman, Seana E Eckels, Umesh Nepali, Aman Reddy, Toren Finkel, Stacey J Sukoff Rizzo

**Affiliations:** ^1^ University of Pittsburgh, Pittsburgh, PA USA; ^2^ 100 Technology Drive, 100 Technology Drive, PA USA; ^3^ University of Pittsburgh School of Medicine, Pittsburgh, PA USA

## Abstract

**Background:**

Studies investigating mTOR signaling provide compelling and reproducible evidence of the extension of lifespan across model organisms by treatment with the mTOR inhibitor rapamycin, and preclinical data suggests neuroprotective benefits of rapamycin in models of Alzheimer’s disease (AD). Rapamycin has potent immunosuppressive and autophagy activating effects though it remains unknown whether rapamycin’s neuroprotective and lifespan enhancing effects are achieved through modulating systemic inflammation, augmenting autophagy, or via some combination of modifying both these factors. Relatedly, the cellular and molecular mechanisms that contribute to rapamycin’s neuroprotective effects in AD remain unclear. The purpose of these studies is to identify the brain‐specific cell types that contribute to the protective effects of mTOR inhibition in progressive AD.

**Method:**

For these studies we crossed a transgenic mouse model of familial early onset amyloid plaque deposition (5XFAD; JAX#034848) with a mouse genetically engineered with reduced mTOR expression to ∼25% of WT levels (mTOR^Δ/Δ^). Previous studies have demonstrated that these mTOR hypomorph mice exhibit a 20% extension in lifespan. We then generated chimeric mice by crossing mTOR^Δ/Δ^/5XFAD carriers to neuronal‐, microglial‐, and astrocyte‐specific Cre+ lines, resulting in mTOR expression reduced to 25% of WT except in the specific Cre+ cell type. Male and female subjects were evaluated longitudinally across the lifespan for measures of healthspan using a frailty assessment and compared to WT littermate controls.

**Result:**

Offspring from all crosses of mTOR^Δ/Δ^; 5xFAD; and neuronal (Syn1‐cre; JAX#003966), astroglial (Gfap‐cre; JAX#012896) or microglial (Cx3cr1‐cre; JAX#025524) cre‐expressing mice were viable with cohorts bred to N = 10/sex/genotype/cre line for longitudinal lifespan and healthspan assessments. Evaluation of frailty scores assessed at 12 months of age reveal the expected increase in frailty in 5XFAD relative to WT littermate controls with reduced frailty scores in mTOR^Δ/Δ^ carrier mice including mTOR^Δ/Δ^/5XFAD crosses.

**Conclusion:**

The present interim results confirm and extend the protective effects of ubiquitous reduction of mTOR expression. Ongoing studies in Cre+ lines will determine in which cell types reduced mTOR signaling is necessary for neuroprotection by comprehensive characterization of these models in functional assessments, AD biomarkers and relevant pathology.

